# Metabolomics Analysis Provides New Insights Into the Molecular Mechanisms of Parasitic Plant Dodder Elongation *in vitro*

**DOI:** 10.3389/fpls.2022.921245

**Published:** 2022-06-20

**Authors:** Yuexia Zhang, Yushi Zhang, Linjian Jiang, Zhaohu Li, Mingcai Zhang

**Affiliations:** ^1^College of Agronomy and Biotechnology, China Agricultural University, Beijing, China; ^2^Shenzhen Institute of Synthetic Biology, Shenzhen Institute of Advanced Technology, Chinese Academy of Sciences, Shenzhen, China; ^3^College of Plant Protection, China Agricultural University, Beijing, China

**Keywords:** dodder, *in vitro*, metabolomics, shoot growth, carbohydrates, parasitic plants

## Abstract

Dodder (*Cuscuta* spp.) species are obligate parasitic flowering plants that totally depend on host plants for growth and reproduction and severely suppress hosts’ growth. As a rootless and leafless plant, excised dodder shoots exhibit rapid growth and elongation for several days to hunt for new host stems, and parasitization could be reestablished. This is one unique ability of the dodder to facilitate its success in nature. Clearly, excised dodder stems have to recycle stored nutrients to elongate as much as possible. However, the mechanism of stored nutrient recycling in the *in vitro* dodder shoots is still poorly understood. Here, we found that dodder is a carbohydrate-rich holoparasitic plant. During the *in vitro* dodder shoot development, starch was dramatically and thoroughly degraded in the dodder shoots. Sucrose derived from starch degradation in the basal stems was transported to the shoot tips, in which EMP and TCA pathways were activated to compensate for carbon demand for the following elongation according to the variations of sugar content related to the crucial gene expression, and the metabolomics analysis. Additionally, antioxidants were significantly accumulated in the shoot tips in contrast to those in the basal stems. The variations of phytohormones (jasmonic acid, indole-3-acetic acid, and abscisic acid) indicated that they played essential roles in this process. All these data suggested that starch and sucrose degradation, EMP and TCA activation, antioxidants, and phytohormones were crucial and associated with the *in vitro* dodder shoot elongation.

## Introduction

*Cuscuta* species (dodders) are holoparasitic plants that can grow on a wide range of dicot plants, such as alfalfa, tomato, and soybean (Dawson, 1989; Lanini and Kogan, 2005). As a rootless and leafless plant, dodder is characterized by the vining growth around host stems to gain access to water and nutrients from hosts *via* a specialized organ termed haustorium (Heide-Jørgensen, 2008; Kim and Westwood, 2015). The parasitic plant, therefore, represents a strong sink for the parasitized host plants. Dodders exhibit severely compromised or no photosynthetic activity, which is likely to contribute less than 1% of the total carbohydrates needed by dodders (van der Kooij et al., 2000; Revill et al., 2005), so they mainly obtain carbohydrates from their plant hosts to maintain growth. On the contrary, dodder shoots may naturally break off from hosts due to physical forces (Singh et al., 1968; Kelly et al., 2001). These detached shoots do not wither away quickly; instead, they are capable of searching for the host to resume the parasitic lifestyle before exhausting the limited resources (Shimizu and Aoki, 2019). This process is very much similar to dodder seedlings germinated from seeds to hunt for host plants. For either detached dodder shoots or new dodder seedlings, the dodder must grow as long as possible to find a host exclusively depending on stored energy. Thus, efficient resource translocation to support growth is a unique ability for dodder to survive in nature. Nevertheless, the metabolic mechanism of detached dodder shoots is still not well characterized.

In plants, carbohydrates are essential as respiratory substrates for the generation of energy and key components for the biosynthesis of storage and structural polysaccharides, such as starch and cellulose. Carbohydrate synthesis, transport, and utilization are dynamic processes strongly dependent on cell physiology, plant organs, developmental stages, and environmental conditions (Ramon et al., 2008; Morkunas et al., 2012). Moreover, carbohydrates have hormone-like functions as physiological signals, which directly regulate the expression of genes and interact with other signaling pathways, which in turn modulate metabolic responses (Ho et al., 2001; Tran et al., 2007). Plants have developed effective mechanisms of perception and transduction of sugar signals, which involve invertases (*INVs*), sucrose transporters (and other specific sugar receptors), and hexokinase (HXK) (Hanson and Smeekens, 2009; Smeekens et al., 2010). Undernutrition deficiency, before oxidative phosphorylation, Embden Meyerhof Parnas (EMP), Tricarboxylic Acid (TCA), and Pentose Phosphate Pathway (PPP) are the main energy respiration pathways. EMP starts from glucose, which is an end product of starch degradation. In addition, maltose, sucrose, and fructose are also involve in the EMP pathway after conversion to glucose. Since lack of an energy source from host plants, reusing stored carbohydrates is essential for the *in vitro* dodder shoots’ growth and re-parasitizing. However, little systematic information is available regarding the involvement of carbohydrates and other metabolites in modulating the *in vitro* development of dodder shoots.

Due to the regulatory and signaling function, carbohydrates could significantly affect the metabolism and development by controlling the essential metabolic processes required to adapt to various stresses, such as drought, chilling, and nutrition starvation (Rolland et al., 2002; Borek et al., 2011, 2012; Lin et al., 2014). Sugar starvation in plant cells, depending on its duration, could trigger physiological-biochemical changes of varying severity, aiming to maintain respiration and other basic metabolic processes (Azad et al., 2008; Borek et al., 2012). Thus, the priority is the acquisition of energy for cell survival, particularly at the later stage of sugar starvation, even at the expense of organelles except for the nucleus and the mitochondria supplying energy (Morkunas et al., 2012). Meanwhile, under nutrition starvation, the accumulation of free radicals is also intensified, potentially leading to oxidative damage. Correspondingly, specific defense strategies are initiated, such as the activation of the enzymatic antioxidant system and phytoferritin accumulation to alleviate oxidative stress (Morkunas and Bednarski, 2008). Additionally, the plant’s ability to respond to nutritional stress generally integrates with internal developmental programs controlled directly by hormones (Rolland et al., 2006; Hammond and White, 2008; Agulló-Antón et al., 2010). Although basic physiological activities of various phytohormones in higher plants are well described, the information on their contents and the possible role in the *in vitro* dodder growth are very limited.

In addition, metabolomics, as an important tool to study the metabolic pathways of the biological system, may provide a new perspective for the understanding of dodder shoot development *in vitro*. Here, the temporal and spatial distribution characteristics of carbohydrates were determined in the *in vitro* dodder shoots, and expression profiles of carbohydrate metabolism-related genes were also detected. Meanwhile, metabolic changes of *in vitro* dodder shoot development were analyzed through the metabolomics data, suggesting that differential metabolites are involved in various metabolic pathways, such as carbon metabolism, secondary metabolite synthesis, oxidative stress, and hormone metabolism. Furthermore, the contents of plant hormones, including abscisic acid (ABA), jasmonic acid (JA), and indole-3-acetic acid (IAA), were first quantified during the *in vitro* dodder shoot growth in this study. These results would provide valuable information for preventing the *in vitro* dodder attack and source-sink partitioning in plants.

## Materials and Methods

### Plant Material

**FIGURE 1:**
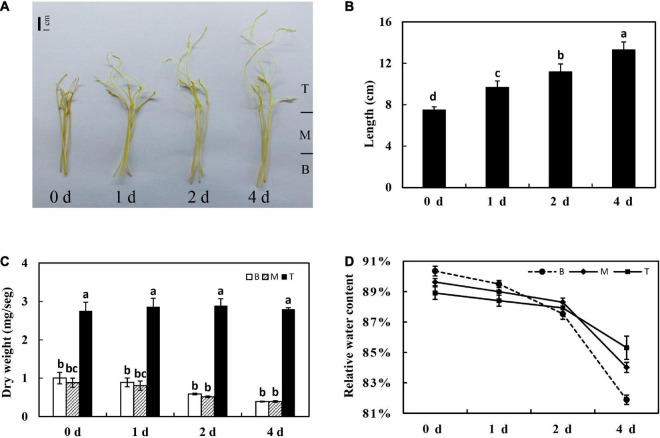
Physiological characteristics of the *in vitro* dodder shoots growth. Morphology **(A)**, length **(B)**, shoot dry weight kinetics **(C)**, and relative water content **(D)** of *in vitro* dodder shoots on days 0, 1, 2, and 4 after excised from host (B, basal stems; M, middle stems; T, shoot tips). Vertical bars are standard deviation of the mean (*n* = 3). Bars marked with the same letter represent no significant difference between each other protected with Fisher’s least significant difference (LSD) at *P* < 0.05.

Seeds of dodder (*Cuscuta suaveolens*, a gift from Dr. P. Reisen, Forage Genetics International, Indiana) were treated with sulfuric acid for 30 min to break seed dormancy, and then sulfuric acid was removed by rinsing with water. Seeds were kept at 23°C for several days on wet filter paper until the seedlings were approximately 3 cm long. Dodder seedlings were placed at the base of 1-week-old alfalfa seedlings. To facilitate parasitization, dodder radicles were slightly submerged in the soil to anchor the host seedlings. A total of 10 dodder seedlings in each pot succeeded in twisting around the stems of alfalfa seedlings and developing haustoria. The day/night length was 16 h/8 h, and the temperature was maintained at 23°C (day) and 18°C (night) in the greenhouse of China Agricultural University, Beijing. After parasitization for 3 weeks, healthy dodder shoot tips (about 7 cm long) were collected and used in this study. Detached shoots were grown in a box containing a layer of wet gauze without any nutrition supplement and incubated in a growth chamber in dark at 23°C. Later on, the detached dodder shoots were harvested on different days (0, 1, 2, and 4 days) after incubation. The morphological basal stem (5 cm) of detached dodder shoot was divided into two segments on average, and the shoot tip (apical bud) was defined as the third segment (Figure 1A), which was used to study the metabolism characteristics of *in vitro* dodder shoot growth.

### Sample Collections

Dodder shoots were harvested on 0, 1, 2, and 4 days after being cultured as described above. The length and fresh weight of harvested dodder shoots were measured. To determine the dry weight and relative water content, samples were oven-dried at 80°C to a constant weight. The fresh samples for gene expression and metabolomic assays were frozen in liquid nitrogen rapidly and stored at –80°C until determination.

### Quantification of Sugar Content

The *in vitro* dodder shoot segments were collected on different days (0, 1, 2, and 4 days) after incubation. The content of starch was determined according to the previous method with some modifications (Hansen and Møller, 1975). Briefly, for measurements of soluble sugar contents including glucose, fructose, and sucrose, fresh samples (approximately 100 mg freeze-dried powder) of *in vitro* dodder shoot segments were prepared, samples were extracted by distilled water for 30 min and centrifuged at 4°C for 10 min, and the supernatant was filtered through a 0.1-μm microfuge spin filter and analyzed by high-performance liquid chromatography (HPLC). Concentrations of glucose, fructose, and sucrose were determined using individual standards (Klann et al., 1993).

### Total RNA Extraction and qRT-PCR

Total RNA was extracted from dodder shoots using the RNA Simple Total RNA Kit (TIANGEN, China) following the instructions of the manufacturer. RNA concentration was determined by using a Nanodrop spectrophotometer (Thermo Fisher Scientific, Inc.). Reverse transcription was performed using a PrimeScriptTM RT reagent kit (TaKaRa, Japan). Then, all qRT-PCRs were performed in triplicate using TB Green R Premix Ex Taq™ II (TaKaRa, Japan) in an ABI-7500 fast Real-Time PCR Detection System. A 2^–ΔΔCt^-based process was used to quantify the relative gene expression level. The *EF-1*α gene was chosen as an internal control to normalize the data in this study (Zhang et al., 2014). Three biological replicates were assayed for each sample. The primers used for real-time qPCR amplification were ordered from Beijing Genomics Institute. The sequences of genes used in this study were produced by the transcriptome database of *C. suaveolens* (Jiang et al., 2013). In particular, the neutral invertase *NINVs* had only one contig in the database. The *SPS* had two contigs; the long sequence (3,522 bp) was used for the expression analysis of *SPS*. The other one was a truncated sequence. Due to lack of gene member annotation, we searched the sequence on the NCBI website, finding that it was homologous with *SPS1* of other plant species, and we defined it as *SPS1* in this study. Similarly, *SUS2* and *SUT2* were also defined by their corresponding homologs of other plant species, respectively. The gene names currently given are based on the best aligned homologous genes using the standard databases (nr, etc.). They may change when better genome sequence information of dodder becomes available. The primers used for real-time qPCR amplification are listed in Supplementary Table 1.

### Metabolite Extraction

Sample extraction was performed according to the previous study with some modifications (Dunn et al., 2011). First, approximately 200 mg of freeze-dried sample was extracted with 1 ml of methanol/acetonitrile/water (2:2:1, v/v/v) containing 2 μl of L-2-chlorophenylalanine (1 mg/ml) as an internal standard. After a 30 s vortex, samples were homogenized at 35 Hz for 4 min and sonicated for 5 min (incubated in ice water), which was repeated three times. Samples were incubated at –20°C for 1 h and then centrifuged at 15,000 × *g* for 15 min at 4°C. The supernatant was transferred into a new tube, and the extracts were vacuum-dried. Second, the dried extracts were reconstituted with 500 μl of acetonitrile/water (1:1, v/v) by sonication on ice for 10 min. The extracts were then centrifuged at 15,000 × *g* at 4°C. Finally, the supernatant (about 70 μl) was filtered and transferred to a fresh 2-ml glass vial for LC/MS analysis. Meanwhile, 10 μl of every sample was prepared as a QC sample for UHPLC-QTOF-MS detection.

### LC-MS/MS Analysis

The freeze-dried extracts were used for metabolomic analyses. LC-MS/MS analyses were performed using an UHPLC equipment (Infinity 1290, Agilent Tech., Santa Clara, CA, United States) with a UPLC BEH Amide column (2.1 × 100 mm, 1.7 μm, Waters Corporation, Milford, MA, United States). The mobile phase consisted of 25 mmol/L ammonium acetate and 25 mmol/L ammonia hydroxide in water (pH = 9.75) (A) and acetonitrile (B). The gradient elution procedure was carried out as follows: 0 min, 95% B; 0.5 min, 95% B; 7 min, 65% B; 8 min, 40% B; 9 min, 40% B; 9.1 min, 95% B; and 12 min, 95% B. The injection volume was 2 μl (positive or negative), respectively. The triple TOF-MS (AB Sciex) was used to acquire MS/MS spectra based on an information-dependent acquisition (IDA). In this mode, the full scan survey MS data was continuously evaluated by the acquisition software (Analyst TF 1.7, AB Sciex), which triggered the acquisition of MS/MS spectra depending on the preset parameters. In each cycle, 12 precursor ions with an intensity above 100 were chosen for MS/MS at 30 V collision energy. The condition of the electrospray ionization (ESI) source was set as follows: ion source gas 1 as 60 psi., gas 2 as 60 psi., curtain gas as 35 psi., source temperature as 600°C, and ion spray voltage floating (ISVF) as 5,000 or 4,000 V in positive or negative modes, respectively.

### Data Processing and Differential Metabolite Analysis

Ions with a relative standard deviation (RSD) < 20% in the QC sample analyses were used for further univariate and multivariate statistics (Smith et al., 2006; Saccenti et al., 2014). We performed a series of multivariate pattern recognition analyses, and principal component analysis (PCA) was first used to initially visualize the differences between the groups. To filter out the unrelated orthogonal variables and obtain the more reliable metabolite information between groups, the orthogonal projection to latent structures-discriminate analysis (OPLS-DA) was used to analyze all comparison groups. In this mode, the differential metabolites were screened by the Student’s *t*-test (*P*-value < 0.05) and the variable importance in the projection (VIP > 1) of the first principal component of the OPLS-DA model. In addition, the KEGG database was used for annotation of the pathways of differential metabolites (DMs). The DMs were used for further analysis and screening of the key pathways based on enrichment analysis and hierarchical clustering analysis.

### Measurements of Hormone Contents

Dodder stems were harvested after growth at different times *in vitro*. LC–MS/MS method with minor modification was used to quantify the phytohormones (Zhang et al., 2020). Briefly, approximately 500 mg of fresh dodder tissue per repeat was ground in liquid nitrogen. First, 200 μl working solution of internal standards (the internal standards of IAA, JA, and ABA were indole-3-acetic-2,2-d2 acid, jasmonic acid-d5, and abscisic acid-d6, respectively) was added to each tube, then 2 ml extraction solvent was added and shook at 4°C for 30 min. Subsequently, 2 ml dichloromethane was added and centrifuged at 15,000 × *g* for 5 min at 4°C, transferring the solvent from the lower phase into a screw-cap vial followed by concentrating through a nitrogen evaporator. The dried extracts were redissolved in 200 μl methanol and then centrifuged at 15,000 × *g* for 10 min at 4°C. The supernatants were transferred to 1.5-ml liquid chromatography (LC) vials and then injected into the LC/MS system.

### Statistical Analysis

Statistical analysis of all data was performed using the SAS software (V8, SAS Institute Inc., United States). Significant differences between treatments were determined by one-way ANOVA followed by Fisher’s least significant difference (LSD) test with a *P*-value < 0.05.

## Results

### Morphological and Physiological Characteristics of the *in vitro* Dodder Shoots Growth

For detached dodder shoots, the basal stems grew slowly and appeared to be senescence; however, the shoot tips continued to grow rapidly, extending about 5 cm long on day 4 compared with that on day 0. The growth rate was highest from 0 to 1 days and then gradually declined from 1 to 4 days (Figures 1A,B). To understand the tissue-specific physiology changes in this process, the shoot was divided into three segments, as shown in Figure 1A. Among the different segments, the dry mass of the basal (B) and middle (M) segments of dodder shoots was reduced by approximately 50% on day 4 after culture *in vitro*. In contrast, the dry weight presented an upward tendency from 0 to 2 days and then decreased from 2 to 4 days in shoot tips (T) (Figure 1C). Meanwhile, the relative water content was significantly decreased in different parts of *in vitro* dodder shoots, especially in the basal stems. In contrast, the shoot tips had higher relative water content compared to other parts (Figure 1D).

### The Variation of Carbohydrates During Dodder Shoots Growth *in vitro*

**FIGURE 2:**
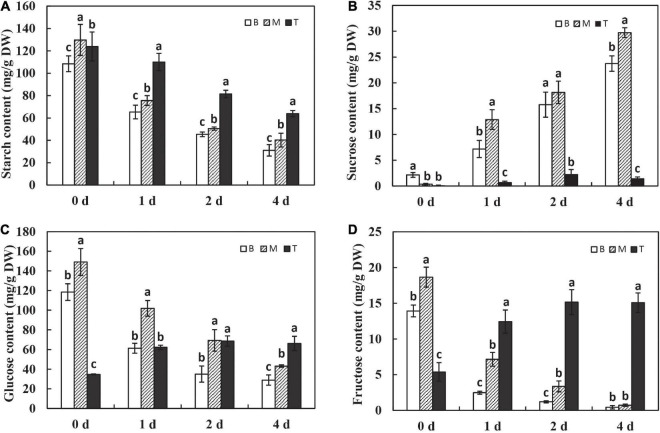
Changes of carbohydrates during dodder shoots growth *in vitro.* The variations of starch **(A),** sucrose **(B),** glucose **(C),** and fructose **(D)** in different segments of *in vitro* dodder shoots (B, basal stems; M, middle stems; T, shoot tips). The values are means, and the vertical error bars are SD (*n* = 3). Different letters at the same time point represent significant difference protected with Fisher’s LSD at *P* < 0.05.

To examine how the *in vitro* dodder shoots recycled stored carbon to maintain the shoot tip development, the contents of starch and soluble sugar were analyzed in this study. During *in vitro* dodder shoot growth, starch content was dramatically decreased on day 1 and then reduced continuously on days 1–4, representing 28.6% and 31.1% of those on day 0 in the basal and middle stems, respectively. A similar pattern was observed in the shoot tips, however, starch content remained at a high level on day 4 in the shoot tips, more than 50% of that on day 0 (Figure 2A). However, sucrose, glucose, and fructose showed the different metabolic patterns between the shoot tips and the basal-middle stems. Sucrose content presented a significant uptrend in the basal and middle stems over the first 4 days *in vitro*. By comparison, sucrose content had a slight increase, peaking on day 2 and then decreasing on day 4 in the shoot tips, which was sustained at a much lower level than that in the basal and middle stems (Figure 2B). Conversely, glucose and fructose contents continuously decreased over time in the basal and middle stems, whereas they were dramatically increased on day 1 and then remained at a higher level in the shoot tips as time progressed *in vitro* (Figures 2C,D).

**FIGURE 3:**
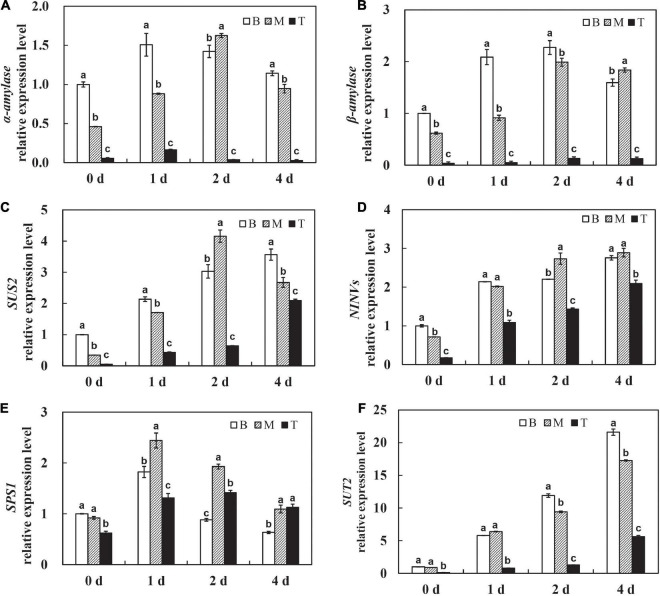
Expression patterns of starch and sucrose metabolism related genes during dodder shoots growth *in vitro.* **(A–F)** Transcript levels of α*-amylase* **(A)** and β*-amylase* **(B)**, *SUS2* **(C)**, *NINVs* **(D)**, *SPS1* **(E)**, and *SUT2* **(F)**, respectively. Bars marked with the same letter represent no significant difference between each other protected with Fisher’s least significant difference (LSD) at *P* < 0.05.

To further investigate the roles of carbohydrates, the expression pattern of amylase, known as the digestive enzyme of starch, was determined. The levels of α*-amylase* and β*-amylase* were significantly upregulated on day 1 after culture *in vitro* and sustained at the high levels with time in the basal and middle stems, whereas the expression of α*-amylase* and β*-amylase* was maintained at a low level in the shoot tips over time (Figures 3A,B). These results explain the dramatic and continuous decrease in starch content in the basal stems, which might be related to the expression of amylases after being isolated from the host. In addition, sucrose, as the primary translocated carbon, needs to be degraded into hexoses for energy supply by either sucrose synthase (SUS) or invertase (INVs) after unloading in sinks (Ruan, 2012). The expression of *SUS2* was significantly induced on the first 2 days in the basal and middle stems, and the shoot tips showed much lower *SUS2* expression levels than other segments on days 0–2 and had a dramatic increase on day 4 (Figure 3C). The *NINVs* (alkaline/neutral invertases genes) irreversibly catalyze the hydrolysis of sucrose into fructose and glucose. The expression of *NINVs* was markedly induced in different segments and remained at a high level on day 4 after culture *in vitro* (Figure 3D). The expression of *SPS1* was upregulated on day 1, and then decreased on days 2–4 in the basal and middle stems. Meanwhile, the expression of *SPS1* fluctuated with a peak on day 2 and was higher in the shoot tips than those in the basal and middle stems on day 4 (Figure 3E). In contrast, the expression of *SUT2* was dramatically upregulated in dodder shoots after culture *in vitro*, especially in the basal and middle stems, and the level in the shoot tips was much lower than that in the other segments during this process (Figure 3F). Taken together, these genes were responsible for the regulation of sucrose metabolism to facilitate the source-to-sink translocation of *in vitro* dodder shoots under nutritional stress.

### Metabolic Profiles in Response to Carbon Stress in the Basal Stems of *in vitro* Dodder Shoots

**FIGURE 4:**
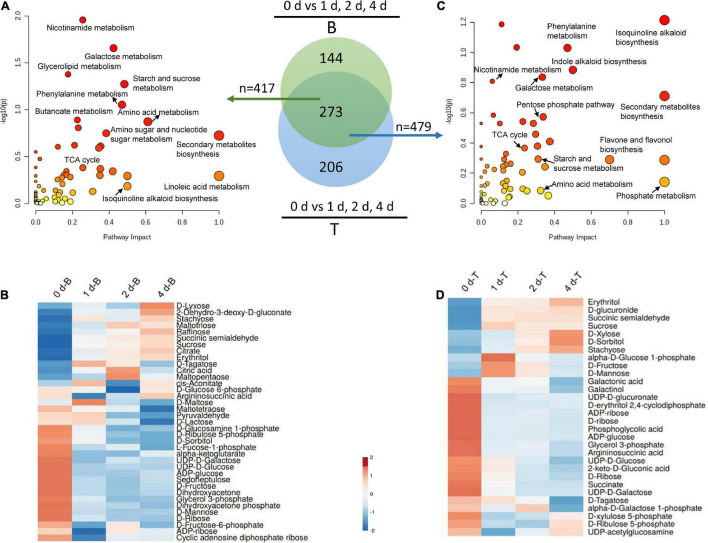
Metabolomics analysis of the basal stems and shoot tips during detached dodder shoots development. **(A,C)** Pathway impact analysis of the basal stems (day 0 vs. day 1, day 2 vs. day 4) **(A)** and shoot tips (day 0 vs. day 1, day 2 vs. day 4) **(C)**, respectively. (B, basal stems; T, shoot tips). The impact indicates the ratio of the number of metabolites mapped to a certain pathway to the total number of metabolites mapped to this pathway. Greater impact factor means greater intensiveness. The *P*-value was calculated using hypergeometric test through Bonferroni correction, and lower *P*-value means greater intensiveness. **(B,D)** Changes of DMs related carbohydrates metabolism in the basal stems **(B)** and shoot tips **(D)**.

To further investigate the regulatory mechanisms of carbon recycling in the basal stems, which acted as an energy source for *in vitro* dodder shoot growth, we examined the metabolomics changes based on a non-targeted UHPLC-QTOF/MS approach to gain a better understanding of the biological changes and adaptive strategies. The Principal Component Analysis (PCA) analysis was employed to evaluate the performance of the UPLC-QTOF/MS. As shown, quality control (QC) samples were separated from tested samples, and the experimental samples were clearly separated into the different subgroups by PC1 and PC2 (Supplementary Figure 1). The results indicated that the UPLC-QTOF/MS approach has good reproducibility. The Orthogonal Projection to Latent Structures-Discriminant Analysis (OPLS-DA, VIP ≥ 1) and the Student’s *t*-test (*P* < 0.05) were applied to detect the differential metabolites (DMs) among subsample groups: day 0 vs. day 1, day 0 vs. day 2, and day 0 vs. day 4 (Supplementary Table 2). Totally, 417 DMs were identified in all three comparisons (Figure 4A) at basal stems. Venn diagrams showed that 121 DMs were shared among these groups (Supplementary Figure 2). Compared with day 0, DMs mainly involved in energy metabolism, secondary metabolism, stress response, and small molecules like amino acids and organic acids. According to the alignment of the KEGG database, the main enrichment pathways of differential metabolites included starch and sucrose metabolism, galactose metabolism, secondary metabolites biosynthesis, and linoleic acid metabolism in the basal stems (Figure 4A). As the *in vitro* dodder shoots were grown in the absence of energy sources from host plants, large numbers of carbohydrate metabolism-related DMs appeared at various levels in response to nutrition stress. We checked that the levels of some compounds, such as sucrose, maltopentaose, maltotriose, raffinose, stachyose, and erythritol, were significantly increased in the basal stems after culture *in vitro* (Figure 4B), suggesting that the massive starch was degraded in the basal stems in this process. Conversely, the TCA cycle, EMP pathway, and pentose phosphate pathway were restrained in this process, which was inferred from the decrease in D-fructose, ADP-glucose, UDP-D-glucose, D-fructose-6-phosphate, D-glucose-6-phosphate, alpha-ketoglutarate, glycerol 3-phosphate, D-ribulose 5-phosphate, and sedoheptulose compared to those in basal stems on day 0 (Figure 4B). In addition, some oxidative stress-related DMs were also detected in this process. For example, glutathione disulfide was decreased on day 0 vs. day 1, S-lactoylglutathione was reduced on day 0 vs. day 1 and day 0 vs. day 4, and L-proline was decreased in all three comparison groups, suggesting an increasing oxidative stress in the basal stems of *in vitro* dodder shoots as time progressed (Supplementary Table 2). It is worth noting that ABA, an important phytohormone regulating stress responses, was greatly induced in the first 2 days *in vitro* and then gradually declined in the basal stems over time (Supplementary Table 2).

### Metabolic Analysis of the *in vitro* Dodder Shoot Tips’ Response to Carbon Stress

The *in vitro* dodder shoot tips acted as a strong sink and required carbon assimilation for growth. To further understand the metabolic regulation mechanism of *in vitro* shoot tips growth, we checked DMs in dodder shoot tips on 0, 1, 2, and 4 days after culture *in vitro*. The number of DMs appeared an increased tendency among day 0 vs. day 1, day 0 vs. day 2, and day 0 vs. day 4 (Supplementary Figure 3, Supplementary Table 3). This indicated that some metabolic pathways were activated by prolonged nutritional starvation. To better understand the metabolite changes caused by different starvation durations in the shoot tips, the Venn diagram of DMs was constructed, showing that 148 metabolites were common among three comparisons, i.e., day 0 vs. day 1, day 0 vs. day 2, and day 0 vs. day 4. Especially, 72 metabolites were unique in day 0 vs. day 4, implying that wider changes happened in this group (Supplementary Figure 3). The DMs were mainly distributed in secondary metabolite synthesis, isoquinoline alkaloid biosynthesis, indole alkaloid biosynthesis, phosphate metabolism, phenylalanine metabolism, carbohydrate metabolism, and so on (Figure 4C).

Additionally, carbohydrate metabolism, such as starch and sucrose metabolism, galactose metabolism, PPP pathway, and TCA cycle, were frequently presented in the enrichment analysis. Hierarchical cluster analyses of these carbohydrate metabolites presented the metabolic characteristics during the *in vitro* dodder shoot tip growth (Figure 4D). Among them, the levels of stachyose, erythritol, D-xylose, and D-sorbitol displayed a significant uptrend in the *in vitro* dodder shoot tips. However, compared with the control (on day 0), the contents of sucrose, D-fructose, and D-mannose were increased on day 1, and then slightly declined on days 2–4. In contrast, the levels of glycerol 3-phosphate, succinate, and D-ribulose 5-phosphate, which are involved in EMP, TCA, and PPP, declined until day 4, indicating that the metabolism slowed down in the *in vitro* shoot tips due to the limited energy supply.

In terms of secondary metabolism, indole alkaloid biosynthesis and isoquinoline alkaloid biosynthesis, especially the IAA (indole-3-acetic acid) biosynthesis pathway, were activated during the *in vitro* dodder shoot tips development. Correspondingly, the levels of L-tryptophan, indoleacrylic acid, and indole-3-lactic acid were greatly increased compared with those on day 0, suggesting that these metabolites might play a critical role in the process of the *in vitro* shoot tips elongating (Supplementary Table 3).

### Comparative Analysis of the Metabolites Between the Basal Stems and Shoot Tips as Time Progressed *in vitro*

**FIGURE 5:**
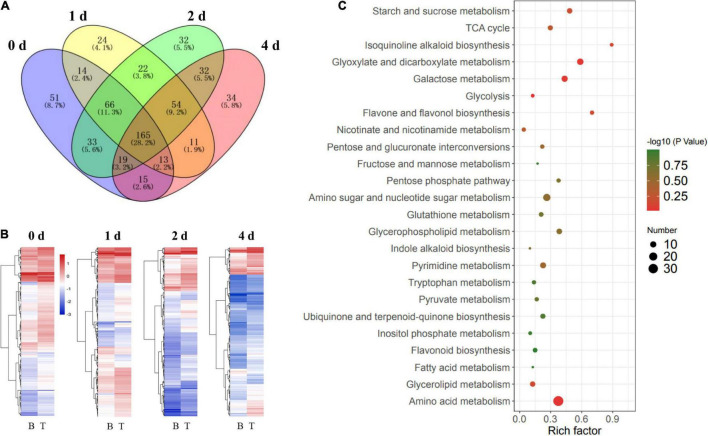
Comparative analysis of metabolites in the *in vitro* dodder shoots at different stages. **(A)** Venn diagram analysis on the time-specific (days 0, 1, 2, and 4) DMs between the basal stems and shoot tips. A total of 165 DMs were shared among four comparison groups. **(B)** The clustering heatmap of DMs in B vs. T on days 0, 1, 2, and 4, respectively (B, basal stems; T, shoot tips). **(C)** Scatter plot of main enrichment KEGG pathways statistics for DMs among four groups. The color and size of the dots represent the range of the –log10 (*P*-value) and the number of DMs mapped to the indicated pathways, respectively.

To gain a better understanding of the source-to-sink translocation in the dodder shoots, metabolite changes were systematically detected by comparative metabolomics analysis of the basal stems and shoot tips during the *in vitro* dodder shoot development. Time-specific (days 0, 1, 2, and 4) DMs were analyzed between the basal stems and shoot tips. Venn diagrams analysis revealed that 165 DMs were commonly regulated between the basal stems and the shoot tips on days 0, 1, 2, and 4 (Figure 5A). Overall, the downregulated DMs showed an increased trend in the *in vitro* dodder shoots as time progressed (Figure 5B). Pathway analysis of DMs acquired from four groups found that the enriched pathways mainly contained sugar metabolism, secondary metabolism, stress response, and amino acid metabolism (Figure 5C), suggesting that these metabolic pathways might play a critical role during the dodder shoot development *in vitro*. In detail, compared to the basal stems, large numbers of DMs involved in EMP, TCA cycle, and pentose phosphate pathways were dramatically induced in the shoot tips on day 0, such as D-fructose 6-phosphate, succinic acid, α-ketoglutarate, glycerol 3-phosphate, phosphoenolpyruvate, D-ribulose 5-phosphate, citrate, glucose 6-phosphate, and UDP-D-glucose (Supplementary Table 4). Subsequently, we checked the DMs between the basal stems and shoot tips during the dodder shoots development on days 1, 2, and 4 *in vitro*. Compared with the basal stems, DMs that were significantly increased in the shoot tips were involved in the EMP, TCA cycle and pentose phosphate pathways, such as D-fructose, D-xylose, alpha-ketoglutarate, glycerol 3-phosphate, D-glucose 6-phosphate, D-fructose-6-phosphate, D-glucose 1-phosphate, D-xylulose 5-phosphate, and D-erythrose 4-phosphate (Supplementary Table 5–7). The result indicated a good energy preparatory status for the rapid growth of the shoot tips. In contrast, some DMs, such as maltotriose, maltopentaose, and raffinose, were continuously upregulated in the basal stems, reaching higher levels than those in the shoot tips on days 1, 2, and 4 *in vitro*. Collectively, these results showed that the carbon metabolic pattern in shoot tips totally differed from that in the basal stems.

Plants will suffer energy stress if sugar availability is insufficient, leading to oxidative stress due to the accumulation of ROS (Apel and Hirt, 2004; Tsukagoshi et al., 2010). Indeed, several antioxidants, including S-lactoylglutathione, glutathione disulfide, L-glutamine, dehydroascorbic acid, and alpha-tocopherol (Vitamin E), were dramatically abundant in the shoot tips compared with the basal stems as time prolonged *in vitro* (Supplementary Table 5-7). Particularly, S-lactoylglutathione, alpha-tocopherol, and glutathione disulfide significantly increased about five folds in the shoot tips compared to those in the basal stems on day 4. These results suggested that robust ROS-scavenging capacity conferred by high antioxidants contents provided crucial support to the rapid elongation of shoot tips.

**FIGURE 6:**
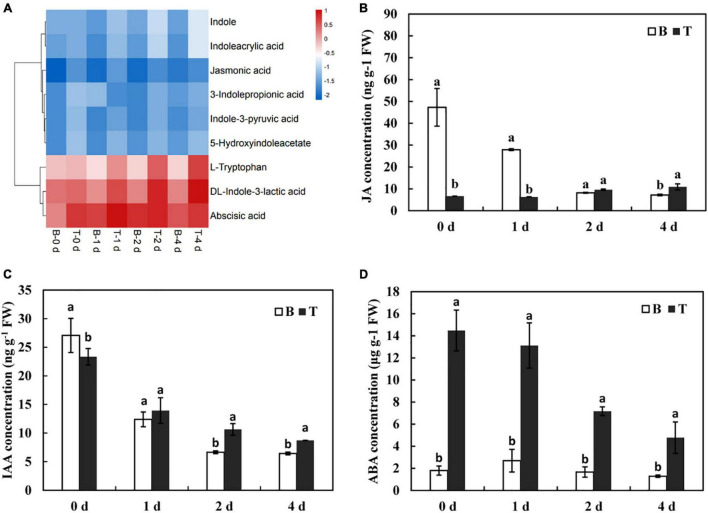
Changes of phytohormone contents during the *in vitro* dodder growth. **(A)** The cluster heatmap of phytohormones-related metabolites by the metabolomics data. **(B–D)** The variations of JA **(B)**, IAA **(C)**, and ABA **(D)** contents in the *in vitro* dodder shoots by LC-MS/MS. Values are means, and the vertical error bars are SD (*n* = 3). Different letters in each panel represent significant difference protected with Fisher’s LSD at *P* < 0.05.

Plant hormones play vital roles in regulating plant growth and development in response to various environmental conditions (Peleg and Blumwald, 2011). In metabolomics analysis, numerous DMs related to the synthesis and signal transduction of hormones, including indole-3-acetic acid (IAA), jasmonic acid (JA), and abscisic acid (ABA), were revealed to have different metabolic patterns between the basal stems and shoot tips. Compared with the basal stems, DMs that involved in IAA metabolism, such as DL-indole-3-lactic acid, indole, L-tryptophan, indole-3-pyruvic acid, and indoleacrylic acid were dramatically increased in the shoot tips as time progressed (Figure 6A), indicating that active IAA metabolism, to some extent, contributed to the elongation of *in vitro* shoot tips. Moreover, the contents of JA and ABA showed an upregulated tendency in the shoot tips compared to those in the basal stems in this process. Subsequently, the contents of JA, IAA, and ABA were also systematically evaluated by LC-MS/MS. JA levels were low from days 0 to 1, and then had a slow increasing tendency and peaked to 10.91 ng/g on day 4 in the shoot tips. In contrast, the contents of JA were dramatically downregulated from days 0 to 1 and reached a lowest level of 7.21 ng/g on day 4 in the basal stems (Figure 6B). Consistent with the variation of JA contents, IAA level continuously decreased from 27.06 ng/g on day 0 to 6.44 ng/g on day 4 in the basal stems, and from 23.34 ng/g on day 0 to 8.21 ng/g on day 4 in the shoot tips, respectively. Nevertheless, the IAA content in the shoot tips was higher than that in the basal stems from days 1 to 4 (Figure 6C). The content of ABA was downregulated in the whole process from 14.48 μg/g on day 0 to 4.77 μg/g on day 4 in the shoot tips, in which the content was much higher than that in the basal stems (Figure 6D). Taken together, the variations of phytohormones indicated they might play essential roles in modulating the *in vitro* dodder shoot development.

**FIGURE 7:**
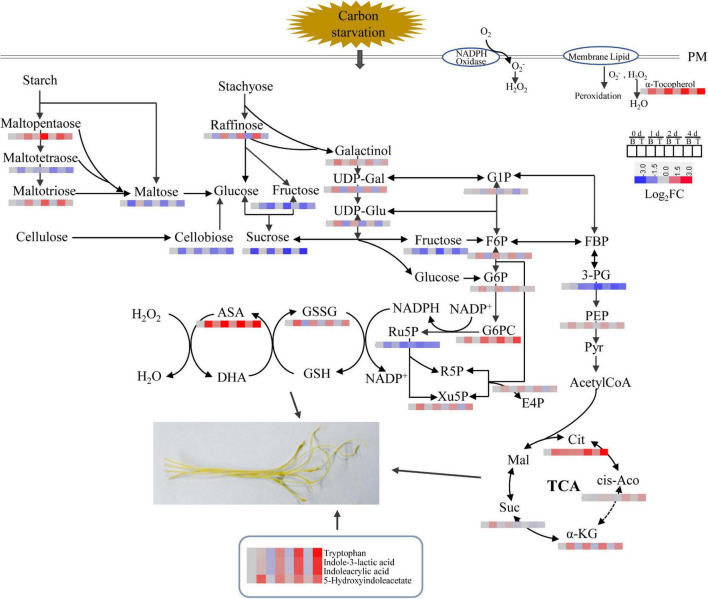
Dynamics of metabolic pathways throughout the *in vitro* dodder shoots development process. The metabolite amounts were shown in heatmaps. PM (plasma membrane), UDP-Gal (UDP-D-galactose), UDP-Glu (UDP-D-glucose), G1P (glucose 1-phosphate), F6P (fructose 6 phosphate), G6P (glucose 6-phosphate), FBP (fructose 1,6 bisphosphate), 3-PG (glycerate-3-phosphate), PEP (phosphoenolpyruvate), Cit (citrate), *cis*-Aco (*cis*-aconitate), α-KG (α-ketoglutarate), Suc (succinate), Mal (malate), G6PC (6-phosphogluconate), E4P (erythose 4-phosphate), R5P (ribose 5-phosphate), Xu5P (xylulose 5-phosphate), GSSG (oxidized glutathione), GSH (reduced glutathione), ASA (ascorbate), and DHA (dehydroascorbate).

Based on the results described above, a schematic representation of the DMs involved in the *in vitro* dodder shoot growth was proposed (Figure 7). Soon after detached from host plants, starch was largely degraded into maltopentaose, maltotetraose, maltotriose, and maltose, leading to the increase in sucrose content in basal stems. Sucrose, the primary translocated carbon, was transferred and then degraded into glucose and fructose in the shoot tips, supplying energy for the following development by activation of EMP, TCA, and PPP pathways. All these data suggested that the efficient recycling of stored carbohydrates was the major contributor to the rapid elongation of the *in vitro* dodder shoots. Meanwhile, phytohormones and antioxidants were also dramatically induced in the shoot tips, indicating their essential roles in this process.

## Discussion

Excised dodder shoots continued growth and elongation for several days to hunt for new host plants. Due to the lack of many genes involved in nitrate, potassium, and phosphate uptake and transporters in the dodder genome and the very limited photosynthetic capability (Sun et al., 2018), the *in vitro* dodder shoots experienced energy stress and mainly relied on their own carbon reserves for development.

In plants, carbohydrate is not only an energetic and structural substrate but also the metabolic intermediate that is used for macromolecule synthesis. Correspondingly, low carbohydrate availability causes a reduction in the rate of growth to retain sufficient carbon for survival (Rolland et al., 2006). Carbon-limiting conditions triggered large numbers of transcriptional responses that led to growth cessation, which included repression of genes involved in metabolism, cell division, and protein repair (Smith and Stitt, 2007). Starch is an integrator of carbon metabolism response; starch turnover and carbon allocation present a central role in the network that coordinates metabolism with growth (Sulpice et al., 2009; Loreti et al., 2018). In this study, starch accounted for about 10% of dry weight, and it was dramatically decreased in the dodder shoots as time progressed, especially in the basal stems (Figure 2A), indicating that starch degradation was highly active and ATP produced by starch metabolism provided sufficient substrate and energy for the following development. The decrease in starch content may be due to the high expressions of α*-amylase* and β*-amylase*, which catalyze the starch hydrolysis in the basal stems (Figures 3A,B). Soluble sugar reflects the basic level of available materials and energy supply. Sucrose, as a kind of starch intermediate product, was higher in the basal stems than in the shoot tips after culture *in vitro* (Figure 2B; Supplementary Table 4–7). In contrast, the contents of glucose and fructose were dramatically increased and sustained at a high level in the shoot tips as time progressed (Figures 2C–D). Sucrose transporter (SUT/SUC) members play vital roles in different biological processes. The role of the *SUT2*/*SUC3* transporter has been implicated in sucrose retrieval along the phloem path (Barth et al., 2003). The strong induction of the sucrose transporter *SUT2* in the basal and middle stems compared to that in the shoot tips indicated its role associated with long-distance phloem transport (Figure 3F). Taken together, we speculated that sucrose, derived from starch degradation in the basal stems, was transported to the shoot tips and degraded into glucose and fructose, which could be rapidly utilized for the *in vitro* shoot tip growth. Therefore, sucrose might be acted as an energy center to ensure carbon supply in this process. In addition, we found that the sucrose content in the basal stems fluctuated over time according to the physiological experiment and metabolomics data (Figure 2B; Supplementary Table 4–7). The detailed regulatory mechanism of sucrose needs to be further studied. Moreover, the metabolomics data also showed that D-maltose, maltotriose, and maltopentaose were upregulated in the basal stems over time, whereas the contents of D-glucose 6-phosphate, alpha-ketoglutarate, D-xylulose 5-phosphate, D-erythrose 4-phosphate, UDP-D-glucose, UDP-D-galactose, and 6-phosphogluconic acid were at higher levels in the shoot tips compared to those in basal stems (Figure 7, Supplementary Table 4–7). These results further indicated that starch was largely broken down to produce enough substances and energy for the following development. Concurrently, the EMP, TCA and pentose phosphate pathways pathway were activated and directly provided carbon demand for the *in vitro* dodder shoot tips elongation.

The plasticity of plant growth is modulated by the complex interplay of sugar and hormones (León and Sheen, 2003; Rolland et al., 2006). DMs related to hormones metabolism, including indole-3-acetic acid (IAA), jasmonic acid (JA), and abscisic acid (ABA), were detected by the metabolomics data (Figure 6A). These results prompted us to systematically examine the dynamic changes of these hormones contents and assess their possible role during the *in vitro* dodder growth. The auxin transport hypothesis suggested that polar auxin transport in the stem was required for bud outgrowth (Li and Bangerth, 1999). In this study, the content of IAA in the shoot tips was higher than that in the basal stems with time, which implied a positive role of IAA during the *in vitro* dodder shoot growth (Figure 6C). Jasmonic acid (JA), a growth-regulating substance, was initially identified as a stress-related hormone in plants. Numerous studies have demonstrated that plant growth and development were regulated in a coordinated manner by the JA and IAA signal transduction pathways, respectively (Wang et al., 2020). In our results, JA content was slowly increased in the shoot tips, whereas they appeared to be a continuous downtrend in the basal stems (Figure 6B). Abscisic acid is an important phytohormone for stress signals (Cutler et al., 2010), and additionally, ABA may regulate sugar transport by influencing the expression of sugar transporter genes (Oliver et al., 2007). Here, we found that ABA content decreased in dodder shoots but maintained a much higher level in shoot tips than that in the basal stems all time (Figure 6C). We deduced that there may be a feedback effect between altered sugars and ABA contents. The functions of these phytohormones need to be further studied in the future.

Plant ROS plays a crucial signaling role in plant response to environmental stress, but the excess ROS could cause oxidative injury to cell components (Morkunas et al., 2003; Gill and Tuteja, 2010). In our previous study, we checked the changes in cell structure, the distribution characteristics of H_2_O_2_, and the activities and expressions of antioxidant enzymes in *in vitro* dodder shoots, suggesting that ROS presented different patterns in the basal stems and shoot tips (Zhang et al., 2021). The metabolomics data further confirmed that the ROS-scavenging antioxidants played an essential role in the *in vitro* dodder shoot elongation and development, which was mainly reflected in the levels of several antioxidants, such as S-lactoylglutathione, glutathione disulfide, dehydroascorbic acid, and alpha-tocopherol (Vitamin E). All of these antioxidants were dramatically upregulated in the shoot tips in contrast to those in the basal stems as time was prolonged *in vitro* (Supplementary Table 4–7). These results implied that the high levels of antioxidants in the shoot tips were important for the *in vitro* dodder shoot elongation by maintaining a robust ROS-scavenging capacity.

## Conclusion

**FIGURE 8:**
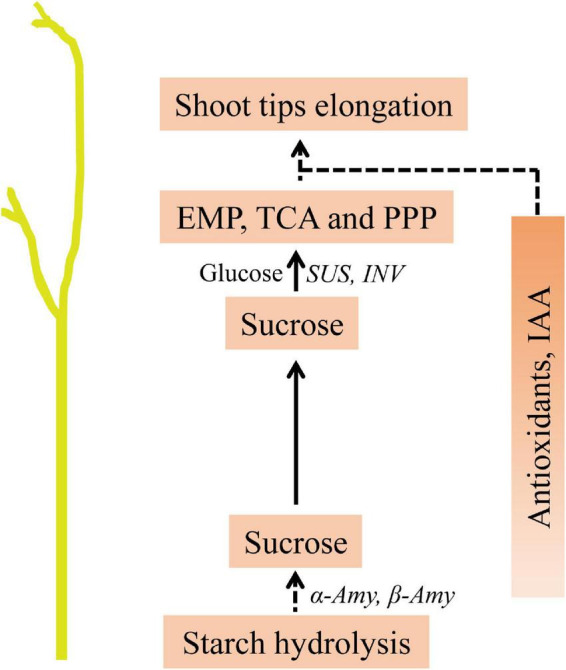
Predicted model of metabolites in modulating the *in vitro* dodder shoot tips elongation. Starch degradation in the basal stems provided the main energy source. Sucrose, derived from starch hydrolysis, was transferred to the shoot tips, in which EMP, TCA and PPP activation directly provide energy and material basis for the following development. Meanwhile, the accumulations of antioxidants and hormone, such as IAA, might play essential roles in this process.

In this study, we analyzed the changes in carbohydrate contents, as well as the gene expression patterns during the *in vitro* dodder shoot growth. Based on these results, we proposed that soluble sugars derived from starch degradation are transported to the shoot tips, supporting the subsequent development of the *in vitro* dodder shoots. Furthermore, we systematically investigated the metabolic changes during the *in vitro* dodder shoot growth, and large numbers of DMs were identified in this process. When the shoot tips gradually developed, starch content dramatically decreased, and the TCA, EMP, and PPP pathways related to DMs were upregulated in the shoot tips, providing energy and substances for the following growth. Meanwhile, antioxidants, such as α-naphthylamine and ascorbic acid, remained at a higher level in the shoot tips than those in the basal stems, indicating the robust ROS-scavenging capacity in the shoot tips. Additionally, we also analyzed phytohormone changes in detached dodder shoots, suggesting their possible roles in modulating the *in vitro* dodder shoot growth. In summary, the large amounts of carbon degradation and transportation, antioxidants in cooperation with phytohormones, contribute to the *in vitro* dodder shoot growth under nutrition starvation (Figure 8). This study would provide basic and systematic information for the detached dodder shoot development.

## Data Availability Statement

The original contributions presented in the study are included in the article/Supplementary Material, further inquiries could be directed to the corresponding author.

## Author Contributions

YXZ and MZ designed the project and oversaw the project management. YXZ and YSZ performed the experiments. YXZ analyzed the data and wrote the draft. YXZ, LJ, ZL, and MZ revised the manuscript. All authors approved the final manuscript.

## Conflict of Interest

The authors declare that the research was conducted in the absence of any commercial or financial relationships that could be construed as a potential conflict of interest.

## Publisher’s Note

All claims expressed in this article are solely those of the authors and do not necessarily represent those of their affiliated organizations, or those of the publisher, the editors and the reviewers. Any product that may be evaluated in this article, or claim that may be made by its manufacturer, is not guaranteed or endorsed by the publisher.

## Supplementary Material

Supplementary Figure 1 PCA plot showing the metabolomic trajectory of the detached dodder shoots growth on days 0, 1, 2, and 4 (B: basal stems; T: shoot tips). QC, quality control samples.

Supplementary Figure 2 Venn diagram of metabolites in the basal stems among day 0 vs. day 1, day 0 vs. day 2, and day 0 vs. day 4.

Supplementary Figure 3 Venn diagram of metabolites in the shoot tips among day 0 vs. day 1, day 0 vs. day 2, and day 0 vs. day 4.

Supplementary Table 1 List of primers used in this study.

Supplementary Table 2 Metabolites identified by LC-ESI-MS/MS in the basal stems during the *in vitro* dodder shoots growth on days 0, 1, 2, and 4.

Supplementary Table 3 Metabolites identified by LC-ESI-MS/MS in shoot tips during the *in vitro* dodder shoots growth on days 0, 1, 2, and 4.

Supplementary Table 4 Metabolites identified by LC-ESI-MS/MS in dodder shoots (basal stems vs. shoot tips) on day 0 *in vitro.*

Supplementary Table 5 Metabolites identified by LC-ESI-MS/MS in dodder shoots (basal stems vs. shoot tips) on day 1 *in vitro.*

Supplementary Table 6 Metabolites identified by LC-ESI-MS/MS in dodder shoots (basal stems vs. shoot tips) on day 2 *in vitro.*

Supplementary Table 7 Metabolites identified by LC-ESI-MS/MS in dodder shoots (basal stems vs. shoot tips) on day 4 *in vitro.*
