# Supplementary material for: Metabolomics Analysis Provides New Insights Into the Molecular Mechanisms of Parasitic Plant Dodder Elongation in vitro
Source: Front Plant Sci. 2022 Jun 20;13:921245. doi: 10.3389/fpls.2022.921245 (PMC9251578; doi:10.3389/fpls.2022.921245)
Supplement: Supplementary Figure 1 — PCA plot showing the metabolomic trajectory of the detached dodder shoots growth on days 0, 1, 2, and 4 (B: basal stems; T: shoot tips). QC, quality control samples. [file Data_Sheet_1.zip › Supplementary Material Presentation - Copy/Supplementary Figtures.docx]

**
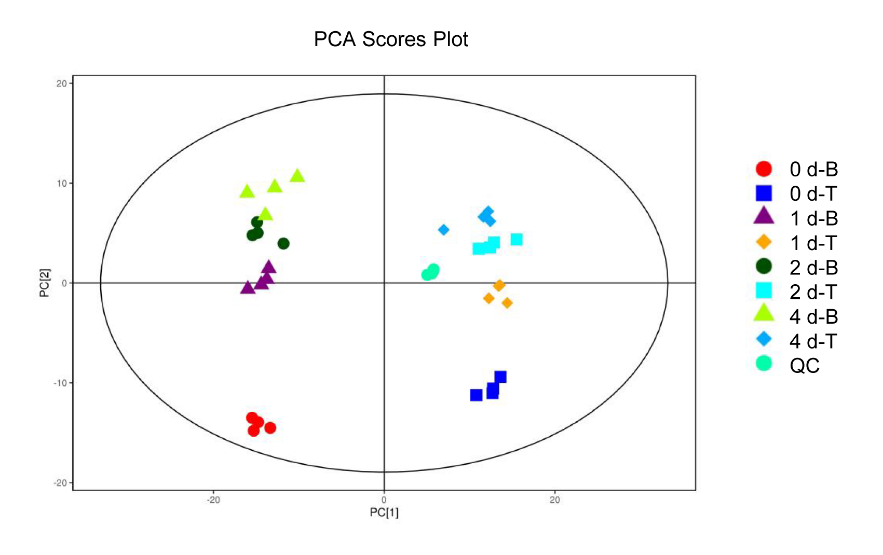
Supplementary Figure S1.** PCA plot showing the metabolomic trajectory of the detached dodder shoots growth at 0 d, 1 d, 2 d and 4 d (B: basal stems; T: shoot tips)*.* QC, quality control samples.


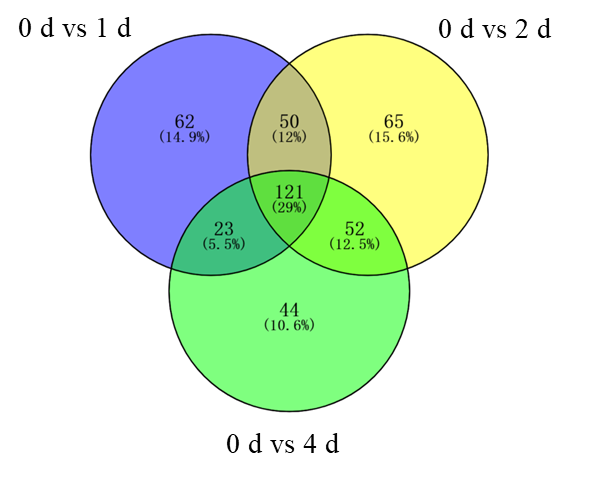


**Supplementary Figure S2.** Venn diagram of metabolites in the basal stems among 0 d vs 1 d, 0 d vs 2 d, 0 d vs 4 d.


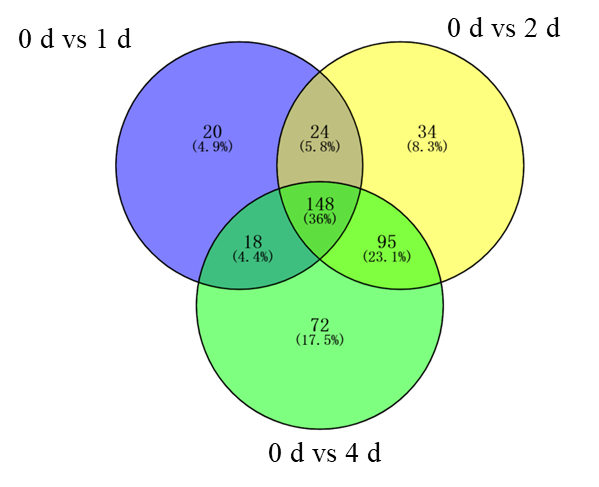


**Supplementary Figure S3.** Venn diagram of metabolites in the shoot tips among 0 d vs 1 d, 0 d vs 2 d, 0 d vs 4 d.
